# A Set of Structural Features Defines the *Cis*-Regulatory Modules of Antenna-Expressed Genes in *Drosophila melanogaster*

**DOI:** 10.1371/journal.pone.0104342

**Published:** 2014-08-25

**Authors:** Yosvany López, Alexis Vandenbon, Kenta Nakai

**Affiliations:** 1 Department of Computational Biology, Graduate School of Frontier Sciences, The University of Tokyo, Chiba, Japan; 2 Human Genome Center, The Institute of Medical Science, The University of Tokyo, Tokyo, Japan; 3 Immunology Frontier Research Center, Osaka University, Osaka, Japan; Max Delbrück Center for Molecular Medicine, Germany

## Abstract

Unraveling the biological information within the regulatory region (RR) of genes has become one of the major focuses of current genomic research. It has been hypothesized that RRs of co-expressed genes share similar architecture, but to the best of our knowledge, no studies have simultaneously examined multiple structural features, such as positioning of *cis*-regulatory elements relative to transcription start sites and to each other, and the order and orientation of regulatory motifs, to accurately describe overall *cis*-regulatory structure. In our work we present an improved computational method that builds a feature collection based on all of these structural features. We demonstrate the utility of this approach by modeling the *cis*-regulatory modules of antenna-expressed genes in *Drosophila melanogaster*. Six potential antenna-related motifs were predicted initially, including three that appeared to be novel. A feature set was created with the predicted motifs, where a correlation-based filter was used to remove irrelevant features, and a genetic algorithm was designed to optimize the feature set. Finally, a set of eight highly informative structural features was obtained for the RRs of antenna-expressed genes, achieving an area under the curve of 0.841. We used these features to score all *D. melanogaster* RRs for potentially unknown antenna-expressed genes sharing a similar regulatory structure. Validation of our predictions with an independent RNA sequencing dataset showed that 76.7% of genes with high scoring RRs were expressed in antenna. In addition, we found that the structural features we identified are highly conserved in RRs of orthologs in other *Drosophila* sibling species. This approach to identify tissue-specific regulatory structures showed comparable performance to previous approaches, but also uncovered additional interesting features because it also considered the order and orientation of motifs.

## Introduction

Understanding the biological information encoded in RRs of genes constitutes one of the greatest challenges in genomics. Analysis of regulatory structure can provide important insight into interactions with specific transcription factors (TFs) and can help predict genes that will be expressed in certain tissues, cell types, or physiological conditions.

Several recent studies have revealed interesting details about regulatory structure and TF binding sites (TFBSs). *Cis*-regulatory elements and motif pairs that are bound by interacting proteins have demonstrated the co-occurrence of specific TFBS in some promoters [1]. Many of the genomic regions that are densely bound by TFs have also revealed new binding relationships between factors [2]. Other studies have examined dependencies among TFBSs. For instance, a set of rules to define the presence and pairwise positioning effects of motifs was developed for modeling human and mouse promoters [3]. Novel motif patterns have also been observed in the promoters of co-expressed genes in *Arabidopsis thaliana* [4]. However, none of these studies considered the orientation, pairwise positioning, and order of the motifs within the RR.

Antenna is a sensory organ located in the anterior part of an insect's head. It is usually covered with olfactory receptors able to detect odor particles in the air, and is sometimes used as humidity sensors for detecting changes in vapor water concentrations. The function of antenna has been studied for understanding the receptor-odorant interactions [5] and analyzing the expression profiles of odorant binding proteins [6]. Other studies have also addressed how flies use the sensing of air motion for controlling flight [7]. Given the importance of antenna and that *Drosophila melanogaster* is a well-studied model organism with a large amount of available genomic data to validate new findings, we have chosen the co-expressed genes in *D. melanogaster* antenna for our analysis of RRs.

Quantitative analyses of enhancer activity of different DNA sequences have revealed many cell type-specific *D. melanogaster* enhancer sequence elements [8]. Computational approaches for finding *cis*-regulatory modules using thermodynamic modeling based on *D. melanogaster* TFBS preferences suggest that positional information is highly important and that weak and strong TFBSs contribute equally to regulation of gene expression [9]. Furthermore, a machine-learning framework that integrated TF binding, evolutionarily conserved sequence motifs, gene expression and chromatin modification data was designed to predict putative functions for uncharacterized genes involved in *D. melanogaster* nervous system development [10]. This integrated framework demonstrated a complementarity between physical evidence of regulatory interactions and coordinated expression. Similarly, reporter gene assays have demonstrated organ-specific expression patterns in *D. melanogaster* [11].

Although some solitary TFBSs are potentially functional, most methods intended to identify *cis*-regulatory modules do not take them into consideration. In addition, despite the clear interdependency among TFBSs, no computational method has simultaneously examined positional and structural relationships of different motifs to model the RR of co-expressed genes. In general, details about the regulatory structures responsible for regulating tissue- or condition-specific gene expression are still lacking.

Here we report a novel computational method that incorporates several different structural features (SFs), including motif orientation, order, position relative to the transcription start site (TSS), and pairwise positioning of motifs to wholly describe the regulatory architecture of *D. melanogaster* antenna-expressed genes. Although a previous framework combined some of these SFs [12], it did not consider the order of regulatory motifs, focusing instead on motif discovery.

Since a broad genomic region around the TSS is considered in this work, we will analyze the *cis*-regulatory modules of *Drosophila* genes, rather than only their core promoter region. To avoid confusion, we will define the “regulatory regions” (RR), as regions that comprise not only the *Drosophila* core promoter region but also enhancers located in its proximity.

This analysis initially predicted six motifs in the RR of antenna-expressed genes, three of which appeared to be novel. We then created a feature collection using all of the predicted motifs and removed irrelevant features with a correlation-based filter. The resulting feature set was further optimized with a genetic algorithm (GA), which achieved an area under the curve (AUC) of 0.841 and produced eight features that best characterize the RR of antenna-expressed genes. This final feature set was used to score all the RRs of *D. melanogaster* genes for unknown antenna-expressed genes sharing a similar regulatory structure. Of the 1000 genes with the highest-scoring RRs, RNA sequencing (RNA-seq) data of antenna-related cell types showed that 76.7% of them were expressed in antenna tissue. We next searched for the presence of our SFs across the *Drosophila* lineage and found evidence for their conservation in the RR of orthologs in other sibling species. Finally, we also used a set of *Caenorhabditis elegans* muscle-expressed genes to compare our method to a similar approach [13], thus uncovering relevant SFs related to the order and orientation of regulatory motifs.

## Results

**Figure 1:**
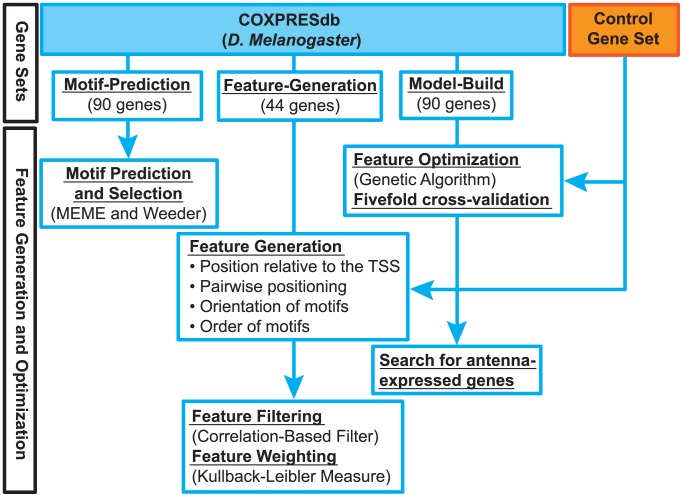
Workflow of our computational method.

Our approach consisted of three main steps (Figure 1): the first step focused on identifying over-represented motifs in the RR of antenna-expressed genes, the second step focused on generating a broad set of SFs and the third step is intended to optimize the set of SFs that best describe the RR of these genes. Co-expression data were obtained for an initial set of 224 *D. melanogaster* antenna-expressed genes from COXPRESdb [14]. The initial set was randomly split into three exclusive subsets: a “motif-prediction” set (90 genes), a “feature-generation” set (44 genes), and a “model-build” set (90 genes).

### Predicted antenna-related motifs

**Figure 2:**
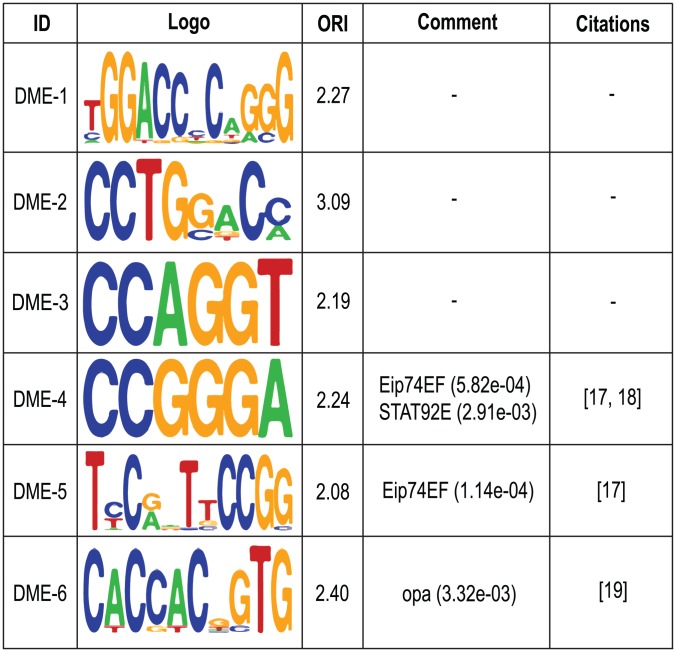
Predicted motifs in RRs of antenna-expressed genes. For each motif, the identifier, logo, and ORI are shown. The known regulatory motif, TOMTOM *p*-value, and citations are also given for motifs that matched already identified motifs.

We first predicted *cis*-regulatory motifs in the 90 RRs (1.5 kbp upstream and 500 bp downstream of the TSS) of antenna-expressed genes in the “motif-prediction” set. We initially uncovered 65 *de novo* motifs. After removal of redundancy in this motif set, 25 non-redundant motifs remained. By using the same “motif-prediction” set, we computed the over-representation index (ORI) [15] for these motifs and removed those with low levels of enrichment in the RRs of antenna-expressed genes. Thus, our final motif collection contained six highly enriched, non-redundant motifs, which we designated *D. melanogaster* enriched (DME) 1–6 (Figure 2). These motifs were compared with those in the JASPAR CORE Insecta database of eukaryotic TF binding profiles [16] and three significant matches were found. DME-4 matched the motif bound by the TFs Eip74EF (ecdysone-induced protein 74EF) and STAT92E (signal transducer and transcription activator), whereas DME-5 and DME-6 matched the motifs bound by the TFs Eip74EF and opa (pair-rule protein odd-paired). To the best of our knowledge, none of these motifs has been reported to be important in antenna. The analysis of acetylation patterns on *Drosophila* ecdysone induced Eip74EF and Eip75B genes has shown acetylation of histone H3 lysine 23 in promoters and its relationship to ecdysone induced gene activation [17]. The activation of STAT92E, a signal transducer in early wing imaginal discs has been shown to inhibit the formation of ectopic wing fields whereas specifies dorsal pleural and inhibits notum identity to divide the body wall [18]. The TF opa1, on the other hand, increases mitochondrial morphometric heterogeneity, thus allowing heart dilation and contractile impairment in *Drosophila* [19]. For the remaining three motifs we did not find any significant match in the JASPAR CORE Insecta database [16], so they appear to be new motifs with potentially important roles in regulating antenna-expressed genes. Comparisons of our six motifs with other previously found in *Drosophila* [20] showed a certain similarity of motifs DME-3 and DME-6 to Motif 7 and Motif 1 (see Table 2 in [20]), respectively.

### Generated and filtered SFs

**Figure 3:**
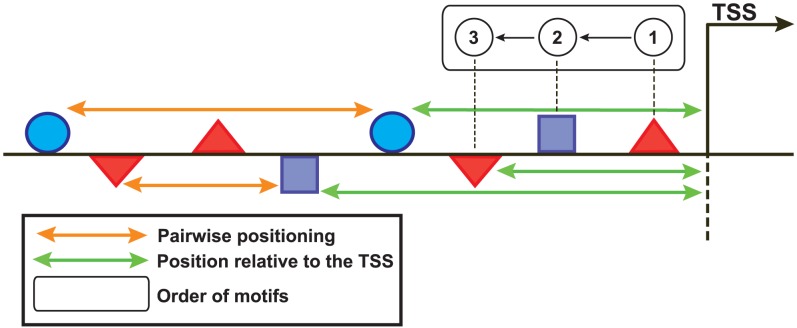
Schematic of our upstream RR-scanning approach. The same approach was followed for the downstream regions. The geometrical forms on and under the black line represent the TFBS on the plus and minus strand, respectively. The orange and green lines and the rectangle indicate the computed SFs.

The six over-represented motifs were used to scan the RRs of genes in the “feature-generation” set for SFs based on position relative to the TSS, pairwise positioning, orientation, and order of these motifs. The regions were scanned in 100-bp windows in both directions (1.5 kbp upstream and 500 bp downstream) from the TSS (Figure 3), and we identified 544 features. To describe the order of motifs, the positions of no more than three motifs were considered per feature. We binarized the features so that each RR was represented as a vector where the presence (1) or absence (0) of each feature was indicated. The 544 features were also examined in the RRs of genes in a negative control set (genes with low expression in antenna; Z-score <−1). We then built a 544×1117 binary matrix (544 features; 44 genes in the “feature-generation” set and 1073 genes in the negative control set). This feature set was filtered with a correlation-based filter [21], which removed any features for which the correlation with the RR of genes in the “feature-generation” set did not predominate, even after removing redundant features. After filtering, 19 SFs remained.

### Optimization of the SFs

**Figure 4:**
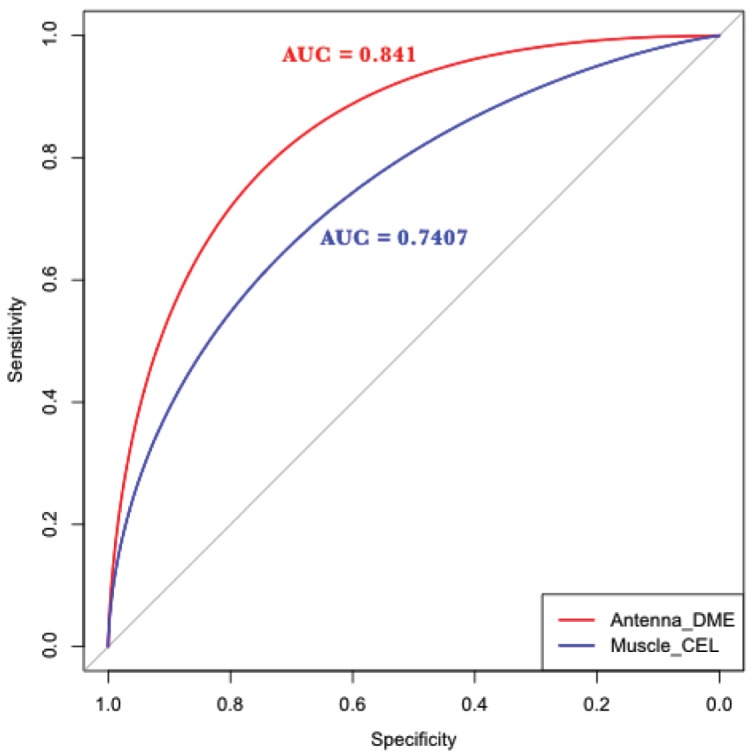
Performance of our GA with two different sets of co-expressed genes. The red line represents the AUC for antenna-expressed genes in *D. melanogaster*, and the blue line represents the AUC for muscle-expressed genes in *C. elegans*.

**Figure 5:**
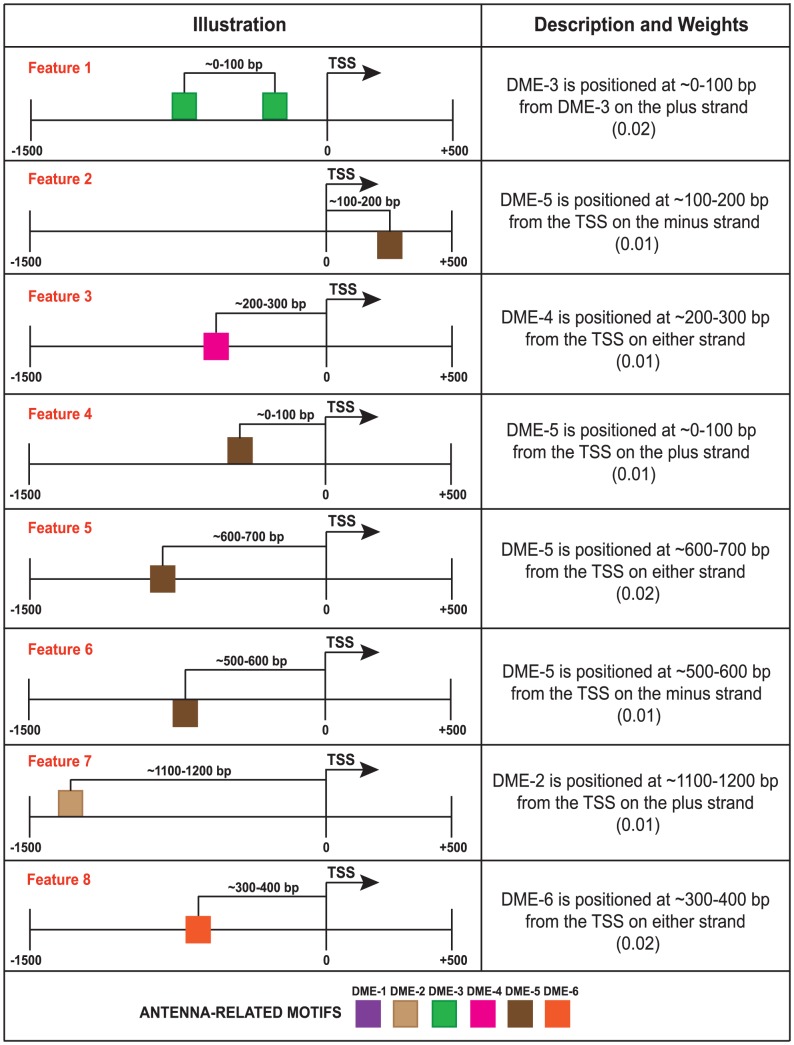
The set of SFs that best describe the RRs of antenna-expressed genes in *D. melanogaster*. For each feature, the relationship between motifs within the feature and the Kullback-Leibler weight are shown. The colored squares represent the antenna-related motifs. Squares on or under the black line indicate motifs on the plus or minus strand, whereas squares in the middle of the black line indicate motifs on either strand.

We weighted the 19 filtered features based on their relevance in describing the RR of antenna-expressed genes and designed a GA to obtain the most informative combination of features. Unlike traditional machine learning methods, GA operates without *a priori* knowledge of the problem to be solved. When used in optimization problems, they tend to be less affected by local maxima than other methods. Because of these advantages, we employed a GA to identify the best combination of features. The “model-build” and negative control gene sets were randomly split into five subgroups, and the GA was trained with four of the subgroups and tested with the remaining one. The fivefold cross-validation (CV) method [22] was repeated 100 times, and the best CV run of the GA, which achieved an AUC of 0.841 (Figure 4), was considered for further analysis. After this validation process, the previous collection of 19 features was reduced to eight high-confidence SFs (Figure 5).

### Genes sharing similar regulatory structure

To evaluate the biological validity of the eight identified SFs, we used them to scan the entire *D. melanogaster* genome for genes with a similar regulatory structure. By using a scoring system that sums up the weight of every present SF, we scored each RR according to the SFs it contained and selected the 1000 genes with the highest-scoring regions. We next obtained the Gene Ontology (GO) terms [23] (uncorrected *p*-value ≤0.01) for these genes and found that a reduced subset of them appear to function in “bristle morphogenesis”, in the biological process that generates sensory bristle structures, or in basal functions of the cell (Table S1). Because the corrected GO term *p*-values were exceptionally high, probably owing to the lack of complete annotation data, we further mapped the RNA-seq data of two cell lines in the third instar larval stage to *D. melanogaster* genome. The cell lines were taken from the tissue eye-antenna disc-derived cell-line (DCCid: modENCODE_4399) and antenna disc-derived cell-line (DCCid: modENCODE_4402), respectively. We found that 7,691 (63.1%) of 12,192 genes in the genome-wide set were expressed in antenna, whereas 767 (76.7%) of 1000 genes with high-scoring RRs according to our identified features were expressed in the antenna-related cell types. From the 7,691 antenna-expressed genes, 5,666 of them were among the 7,691 genes with highest-scoring RRs. This percentage of antenna-expressed genes (76.7%) is given because we have used a high threshold (Fragments Per Kilobase of transcript per Million mapped reads (FPKM)>1) compared to previous studies [24] (FPKM>0.05). Because this expression data originated from immature cells, many receptor genes showed little or no expression at all.

**Figure 6:**
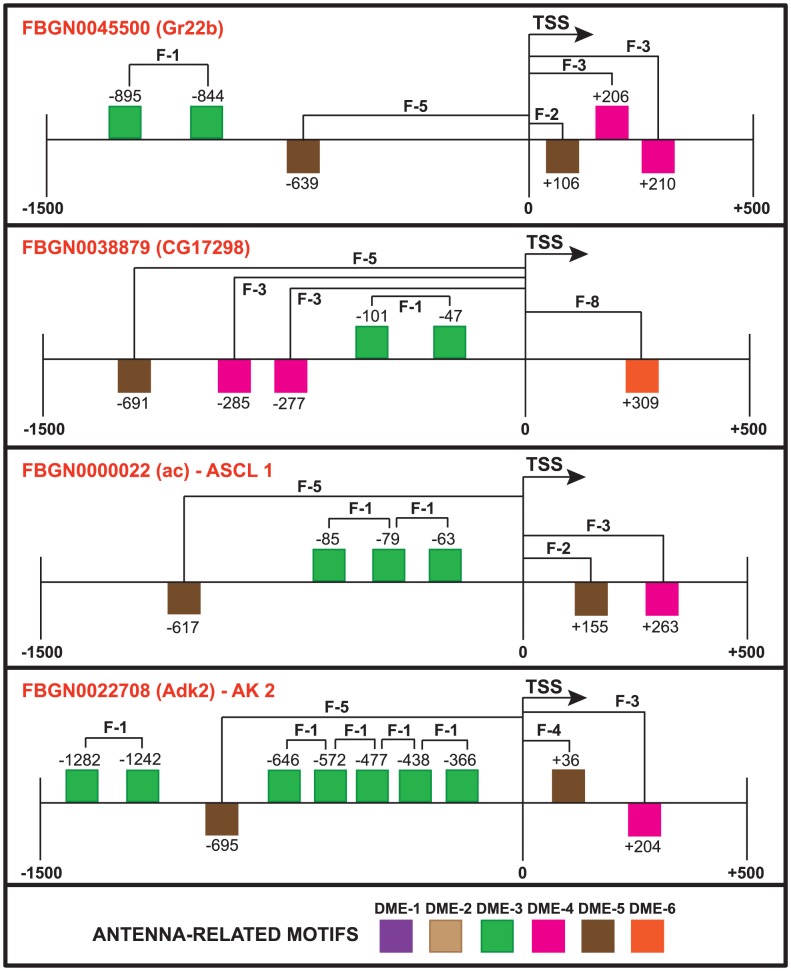
Detailed architecture of four of the highest-scoring *D. melanogaster* RRs. Each ‘F’ represents a SF. The human gene names are shown for the *D. melanogaster* genes with human orthologs.

We noted that among the 50 genes with highest-scoring RRs (Figure S1 and Table S2), only two were also included in the “motif-prediction”, “feature-generation”, and “model-build” sets. Since each gene in the initial sets has different SFs, genes with RRs containing more SFs or more heavily weighted SFs will score higher compared to others. From the initial set of 224 genes, 81 genes were among the 1000 top scoring genes. We next verified how many of the 50 highest-scoring RRs contained the identified SFs. We found that all 50 of the RRs contained DME-3 at ∼0–100 bp from DME-3 on the plus strand (feature 1), 11 RRs had DME-5 at ∼100–200 bp from the TSS on the minus strand (feature 2), 34 RRs had DME-4 at ∼200–300 bp from the TSS on either strand (feature 3), 40 RRs had DME-5 at ∼600–700 bp from the TSS on either strand (feature 5), and 19 RRs had DME-6 at ∼300–400 bp from the TSS on either strand (feature 8). The scoring of *D. melanogaster* RRs uncovered genes with known biological functions in sensory organs and others with unknown biological function. Figure 6 depicts four of the 50 highest-scoring RRs, three of which are involved in detecting chemical stimuli, sensory organ development, and neurogenesis, and one with unknown biological function. *Gr22b* (FlyBase ID FBGN0045500) encodes a protein involved in detecting chemical stimuli [25]. The RR of *Gr22b* shares three SFs 1, 3, and 5 with that of *ac* (FlyBase ID FBGN0000022) and *Adk2* (FlyBase ID FBGN0022708), which encode proteins involved in sensory organ development and neurogenesis [26], [27]. The RR of gene CG17298 (FlyBase ID FBGN0038879) shares the previous three features with that of genes *Gr22b*, *ac* and *Adk2* while also containing feature 8 (Figure 6).

**Figure 7:**
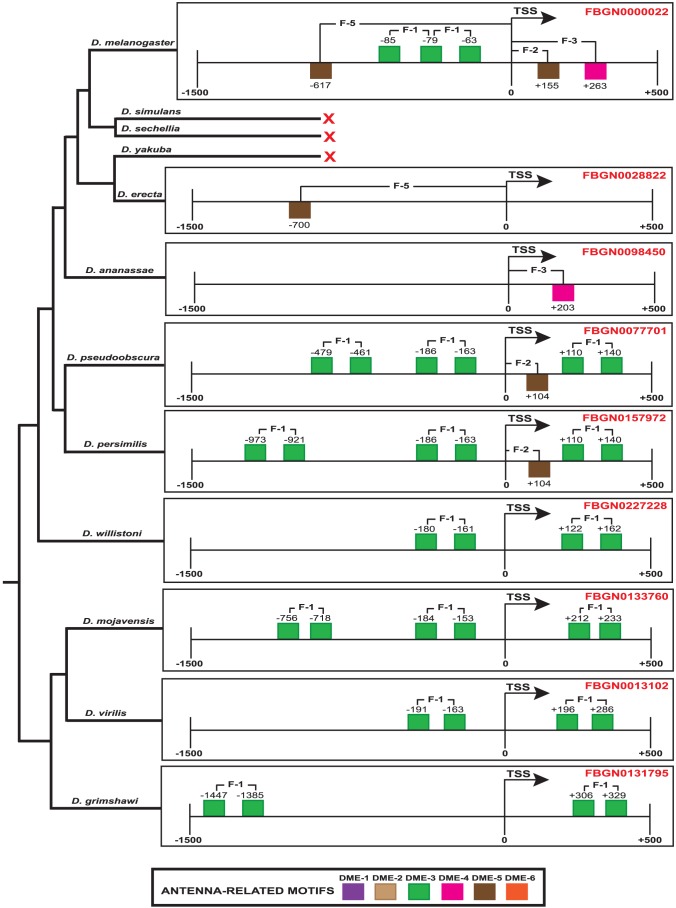
Conservation of SFs between the RR of *D. melanogaster ac* and the RRs of orthologs across the *Drosophila* lineage. The colored squares represent the antenna-related motifs. Squares on or under the black line indicate motifs on the plus or minus strand, respectively. The red cross means either that the respective region does not contain conserved features or that there is no such ortholog. The phylogenetic tree is based on the tree reported in [46].

Furthermore, genomes of 11 *Drosophila* sibling species were downloaded from FlyBase database [28]. RRs of each *Drosophila* specie's genes were extracted. Each RR was scanned for potential binding sites of the six enriched antenna-related motifs. We next scanned every RR for the presence of our eight SFs. As a result, we found that feature 1 is extensively conserved across *Drosophila* orthologs. In addition, the RRs of the closest orthologs mostly share features 2, 3 and 5 (Figure 7 and Figures S2 and S3).

### Comparison with another method

We compared our computational method to a similar reported promoter structure-modeling approach [13] with a set of 121 *C. elegans* muscle-expressed genes. We randomly split such a set into three independent subsets: a “Ce motif-prediction” set (48 genes), a “Ce feature-generation” set (23 genes), and a “Ce model-build” set (50 genes). The *C. elegans* genome was obtained from WormBase [29]. RR extending from 1 kbp upstream to 200 bp downstream of the TSS was analyzed. Two different motif-discovering algorithms: MEME [30] and Weeder [31] were used for predicting *de novo* motifs in the RRs of muscle-expressed genes in the “Ce motif-prediction” set. A total of 64 *de novo* motifs were uncovered, and 18 non-redundant motifs were obtained after removing redundancy. We next computed the ORI [15] of each previous motif, leaving us with 11 over-represented motifs (Table S3). Comparison of the motifs with those in the JASPAR CORE Nematoda database [16] showed that *C. elegans* motifs (CEL) 4, 6, and 9 matched motifs bound by the TFs DAF-12 (protein DAF-12), EOR-1 (protein EOR-1), and DPY-27 (chromosome condensation protein dpy-27), respectively. On the other hand, eight motifs did not significantly match any known motif and were regarded as potentially novel *C. elegans* muscle-related motifs. It has been reported that DAF-16 enhances *daf-12* expression while suppressing *daf-9* expression during larvae formation upon cholesterol starvation [32]. Further, genes *eor-1* and *eor-2* play an important role in promoting terminal neuron differentiation and apoptotic death of the male hermaphrodite neurons [33]. On the other hand, protein DPY-27 condenses the chromatin structure of X chromosome, thus reducing the expression of its genes [34]. To the best of our knowledge, these three TFs DAF-12, EOR-1 and DPY-27 have not been reported to directly regulate muscle-expressed genes.

Comparison of the 11 enriched muscle-related motifs with previously reported motifs has revealed some interesting similarities. Motifs CEL-5 and CEL-6 are similar to Motif 2 and Motif 5 [13] and to M1 [35]. The first six nucleotides of motif CEL-8 seem to be similar to Motif 6 [13] (Table S3 and Figure 4 in [13]). Finally, motif CEL-4 is similar to motif M4 [35] and has also matched DAF-12 like motif M4 (Table 1 in [35]).

All 11 muscle-related motifs were used to scan the RRs of genes in the “Ce feature-generation” set to build a collection of SFs such as motif orientation, order, position relative to the TSS, and pairwise positioning of motifs for describing the RR of *C. elegans* muscle-expressed genes. As a result, 887 SFs were obtained and also analyzed in the RRs of genes in a negative control set. We then filtered the irrelevant SFs with a correlation-based filter [21], leaving us with 13 significant features. We subsequently designed a GA to obtain those highly informative SFs describing the RRs of muscle-expressed genes and five SFs were finally obtained (Table S4). The “Ce model-build” set was split into five subsets for fivefold CV [22]. The GA was trained in four folds whereas the remaining fold was used for validating the performance of the GA. The best CV run of the GA achieved a comparable AUC (0.7407) to that achieved in previous studies [13] (Figure 4) while uncovering SFs that also considered the orientation and order of motifs. The five-feature set was used for scoring all *C. elegans* RRs and identifying unknown muscle-expressed genes with similar regulatory structure. The 50 genes with highly scoring RRs were obtained (Figure S4 and Table S5). We also uncovered two *C. elegans* genes (B0304.1A and F07A5.7A.1) previously reported in a similar analysis [13] (Figure S5). These results illustrate the general applicability of our approach.

## Discussion

In this study, we combined several types of SFs that have not been simultaneously considered in previous approaches aimed at modeling the RR of co-expressed genes. This approach revealed that the orientation and order of regulatory motifs are important features that also need to be taken into account when describing the regulatory structure. Interestingly, we found that although the orientation of motifs in the RR was important to both *D. melanogaster* antenna-expressed genes and *C. elegans* muscle-expressed genes, the order of motifs was only relevant to RRs of the muscle-expressed genes. It suggests a certain degree of interactions or collaborative regulation between the proteins binding these motifs. The correlation-based filter successfully removed irrelevant features, greatly reducing the initial feature space and improving the performance of the GA. For *D. melanogaster* antenna-expressed genes, the most relevant feature set related to pairwise positioning, orientation, and positioning of motifs relative to the TSS. The analysis of gene expression levels using RNA-seq data confirmed that a subset of the antenna-expressed genes indeed share a similar regulatory architecture.

Furthermore, the conservation of these SFs in RRs of orthologs across the *Drosophila* lineage and the fact that more closely related sibling species tended to share more SFs provide strong evidence for the positive selection of these regulatory motifs (Figure 7 and Figures S2 and S3). For example, pairs of DME-3 motifs are positioned at ∼0–100 bp from each other on the plus strand across the *Drosophila* phylogenetic tree, demonstrating the conservation of regulatory motifs among the *Drosophila* sibling species and the ability of our approach to consistently detect these motifs.

Our computational method achieved an AUC comparable to that of a similar approach with *C. elegans* muscle-expressed genes [13], but the SFs we obtained were more detailed and descriptive because they included the important consideration of orientation and order of regulatory motifs. The order of motifs within the RRs of muscle-expressed genes appears to suggest certain interaction or collaborative regulation between the TFs binding such motifs. Our approach also identified genes with known biological functions in *C. elegans* muscle tissue, in which the orientation and order of motifs in their RRs appeared to be important. For instance, the RR of B0304.1A contains CEL-4 at ∼200–300 bp from CEL-8 on opposite strands (feature 2 in Table S4), whereas that of F07A5.7A.1 has CEL-10 at ∼400–500 bp downstream from CEL-4 on the plus strand (feature 4 in Table S4).

## Materials and Methods

Figure 1 shows an overview of the computational methods used in this study.

### Databases

We selected the expression values of *D. melanogaster* genes from COXPRESdb (http://coxpresdb.jp) [14], which contains data for co-expressed genes in multiple organisms. The expression data have been derived from adult antenna (Gene Expression Omnibus accession number GSE27927) and the biological experiment used six pools of flies. Samples were taken at 0, 24 and 48 hours and separated in each body part (antenna, head, body and proboscis tissue) for each pool [36]. The *D. melanogaster* genome (version 5.51) of FlyBase (http://flybase.org) [28] was downloaded. Since COXPRESdb contains expression data for 12,192 *D. melanogaster* genes under many different experimental conditions, we chose only 56 microarrays derived from antenna, head, body, and proboscis tissues (14 available microarrays per tissue). From these microarray data, more than 100 highly expressed genes were selected per tissue as described in [4]. A total of 224 antenna-expressed genes were selected for further analysis. In addition, we computed the Z-score of each gene in antenna and the group of 1073 genes (Z-scores <−1) was designated as the negative control set that contains non-antenna-expressed genes.

### Gene sets

The set of antenna-expressed genes was further split into three non-overlapping subsets: a “motif-prediction” set (Table S6), a “feature-generation” set (Table S7), and a “model-build” set (Table S8). The first subset of 90 genes was employed to predict *de novo* motifs. The second subset of 44 genes was used to generate and filter irrelevant SFs, whereas the third subset of 90 genes was used to obtain the optimal combination of features to describe the RRs of antenna-expressed genes. The TSSs data were downloaded from FlyBase database [28], and the most upstream TSS among a set of alternative TSSs of a gene was regarded. For the analysis of SFs we used the region 1.5 kbp upstream to 500 bp downstream of the TSS [37].

### Prediction and selection of motifs

The motif-discovery algorithms MEME (http://meme.nbcr.net/meme/) [30] and Weeder (http://159.149.160.51/modtools/) [31] were used for motif prediction. We ran MEME [30] to search for 6- to 12-bp motifs using any number of binding sites per sequence on both strands. We used Weeder [31] to search for 6-bp motifs with one mutation, 8-bp motifs with two and three mutations, 10-bp motifs with three and four mutations, and 12-bp motifs with four mutations on both strands. All of the predicted motifs were compared to each other using TOMTOM (http://meme.nbcr.net/meme/tomtom-intro.html) [38] to remove redundant motifs. From each pair of matching motifs with *p*-value ≤0.001 (TOMTOM threshold [38]), we keep the one with the higher information content [39]. We next computed the ORI [15] of every selected motif and chose only those with ORI≥2.0 and compared them to motifs in JASPAR CORE (Insecta/Nematoda) database (http://jaspar.binf.ku.dk) [16]. Motifs that did not significantly match (with *p*-values <0.01) any known motif were regarded as potentially new regulatory motifs.

### Generation of a SF library

To determine the threshold for defining binding sites of each motif, we independently scanned 1000 random RRs and computed the score for each bp in a position-specific scoring matrix. From this score, a threshold of about one binding site in 5000 bp was chosen. Subsequently, the over-represented motifs along with the “feature-generation” and negative control sets were employed to identify a collection of SFs of the RRs. We scanned the RRs of genes in the “feature-generation” set in 100-bp windows for four different types of SFs related to potential motifs, including motif position relative to the TSS, pairwise motif positioning, order, and orientation (Figure 3). For the positioning of motifs relative to the TSS, the 100-bp window was centered at the TSS. To examine the pairwise positioning of motifs, we considered one of the motifs as the starting point. The order of motifs was assessed relative to the TSS independent of motif orientation. These SFs were also examined in RRs of genes in the negative control set. As a result, a binary matrix was established that describes the presence (1) or absence (0) of the features in the RRs of both sets.

### Removal of irrelevant SFs

Our initial feature set was relatively large and contained considerable redundancy of SFs. We therefore introduced a pre-processing filtering step that was not used in [4] to improve the computational efficiency of the GA while eliminating irrelevant and redundant features that might not correctly describe the RR of co-expressed genes. This correlation-based filter [21] has a relatively low computational time and makes use of a measure known as “symmetrical uncertainty”. It thus reduces the feature space by removing those features with low correlation.

### Weighting of SFs

Each feature was weighted based on its importance in describing the RR. Information gain measures have been used for weighting features [40], but this approach does not always describe particular probabilistic events. Therefore, we used the Kullback-Leibler metric [41] as defined below,
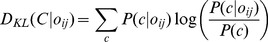
(1)where *P(c)* is the probability of class *c* (the positive class is composed by RRs of genes in the “feature-generation” set, whereas the negative class comprises the RRs of genes in the control gene set), *o_ij_* is the RR with the *j* value (presence/absence) of the SF *i*, *P(c|o_ij_)* is the probability of class *c* given the RRs *o_ij_* and *D_KL_(C|o_ij_)* is the Kullback-Leibler measure of class *C* (the positive and negative classes) given the RRs *o_ij_*. These variables were used to define the weights of each SF as follows,
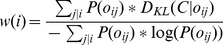
(2)where *w(i)* is the weight of the SF *i*, and *P(o_ij_)* is the probability of the RRs *o_ij_*.

This weighting step gives a higher importance to SFs that are highly present in the RRs of antenna-expressed genes. Finally, the weights are normalized to sum up to 1.

Two kinds of classes were considered: RRs of antenna-expressed genes in the “feature-generation” set (positive class, 1) and RRs of non-antenna-expressed genes in the negative control set (negative class, 0). The weights of the SFs selected by the GA (described below) were used to score the *D. melanogaster* RRs and identify genes with similar regulatory structures. The sum of the weighted scores of each relevant feature within a RR was the final overall score of that region.

### Design of Genetic Algorithm

A GA was designed to obtain the most informative set of SFs. GA is a machine learning algorithm that simulates the genetic evolution process of living organisms at the population or chromosome level [42]. In this context, a chromosome refers to a binary string, which contains different genes (binary characters). In our GA, a chromosome was considered to have as many genes as the number of SFs being assessed (19 features for RRs of antenna-expressed genes), and the fitness proportionate selection method was used to choose the solution (feature arrangement) with the highest fitness value from the “model-build” (positive) and control (negative) gene sets. Even with low probability, this selection method allows choosing solutions with low fitness values that could be important SFs during the recombination process. For convergence, we stopped the algorithm when accuracy has reached 90% or has iterated 10000 times. To validate the GA-derived model, both gene sets were randomly split into five subgroups for fivefold CV [22]. Each of the five CV runs produced a distinct chromosome, and features present in at least three of these chromosomes were considered the best set of SFs.

### Genome-wide search for genes with similar regulatory structures

The *D. melanogaster* genome was scanned for genes with a similar regulatory structure, and the weights of the best features were used to score each *D. melanogaster* RR. The GO terms [23] associated with the identified genes were analyzed to confirm that these genes were related to antenna tissue or to basal cellular functions (Table S1). We downloaded RNA-seq data from two *D. melanogaster* cell lines (eye-antenna disc-derived (DCCid: modENCODE_4399) and antenna disc-derived (DCCid: modENCODE_4402)) and mapped them to the *D. melanogaster* genome (release r5.52) with TopHat (http://tophat.cbcb.umd.edu) [43] and Bowtie (http://bowtie-bio.sourceforge.net) [44]. We next measured gene expression in Fragments Per Kilobase of transcript per Million mapped reads with Cufflinks (http://cufflinks.cbcb.umd.edu) [45], and a relative expression level of >1.0 was used to define the set of expressed genes. We used the above RNA-seq data from immature antenna because no available data for adult antenna was found in the Model Organism Encyclopedia of DNA Elements database (http://www.modencode.org). Finally, we also downloaded the genomes of 11 *Drosophila* sibling species from FlyBase [28] and checked the conservation of our SFs in RRs of orthologs across the *Drosophila* lineage.

## Conclusions

We have developed a new computational approach to describe the RRs of co-expressed genes. The proposed approach offers an advantage over previous methods in that it considers the order and orientation of regulatory motifs. Validation using RRs of *D. melanogaster* antenna-expressed genes identified three potentially novel motifs. Our analysis showed that the orientation and order of motifs are highly relevant in modeling the RRs of co-expressed genes and should be considered in future studies. The SFs we identified are also conserved in RRs of orthologs across the *Drosophila* lineage, further indicating the reliability of our findings. Future work is aimed at incorporating more tissues into our method for determining those SFs that might be either shared or specific to each tissue. We also plan to integrate other interesting features such as the average free energy of DNA and the periodicity to increase our understanding of transcription regulation of co-expressed genes.

## Supporting Information

Figure S1 **The 50 highest-scoring regulatory regions in** ***D. melanogaster*** **.**
(PDF)

Figure S2 **Conservation of structural features between the regulatory region of** ***D. melanogaster Adk2*** **and the regulatory regions of orthologs across the** ***Drosophila*** **lineage.**
(PDF)

Figure S3 **Conservation of structural features between the regulatory region of** ***D. melanogaster Gr22b*** **and the regulatory regions of orthologs across the** ***Drosophila*** **lineage.**
(PDF)

Figure S4 **The 50 highest-scoring regulatory regions in** ***C. elegans*** **.**
(PDF)

Figure S5 **Detailed architecture of two** ***C. elegans*** **regulatory regions uncovered by our method and previously reported [13]**.
(PDF)

Table S1 **Gene ontology terms for the 1000 genes (excluding genes in the initial sets) with the highest-scoring regulatory regions.**
(PDF)

Table S2 **Description of 50** ***D. melanogaster*** **genes with high scoring regulatory regions.**
(PDF)

Table S3 **Predicted motifs in regulatory regions of muscle-expressed genes.**
(PDF)

Table S4 **The set of structural features that best describe the regulatory regions of muscle-expressed genes in** ***C. elegans*** **.**
(PDF)

Table S5 **Description of 50** ***C. elegans*** **genes with high scoring regulatory regions.**
(PDF)

Table S6 **FlyBase IDs of 90 antenna-expressed genes in the “motif-prediction” set.**
(PDF)

Table S7 **FlyBase IDs of 44 antenna-expressed genes in the “feature-generation” set.**
(PDF)

Table S8 **FlyBase IDs of 90 antenna-expressed genes in the “model-build” set.**
(PDF)
