# Supplementary material for: A Set of Structural Features Defines the Cis-Regulatory Modules of Antenna-Expressed Genes in Drosophila melanogaster
Source: PLoS One. 2014 Aug 25;9(8):e104342. doi: 10.1371/journal.pone.0104342 (PMC4143197; doi:10.1371/journal.pone.0104342)
Supplement: Figure S1 — The 50 highest-scoring regulatory regions in D. melanogaster . (PDF) [file pone.0104342.s001.pdf]

Figure S1: The 50 highest-scoring regulatory regions in *D. melanogaster*. Colored squares represent antenna-related motifs. Squares above or under the black line represent motifs on the plus or minus strand, respectively.

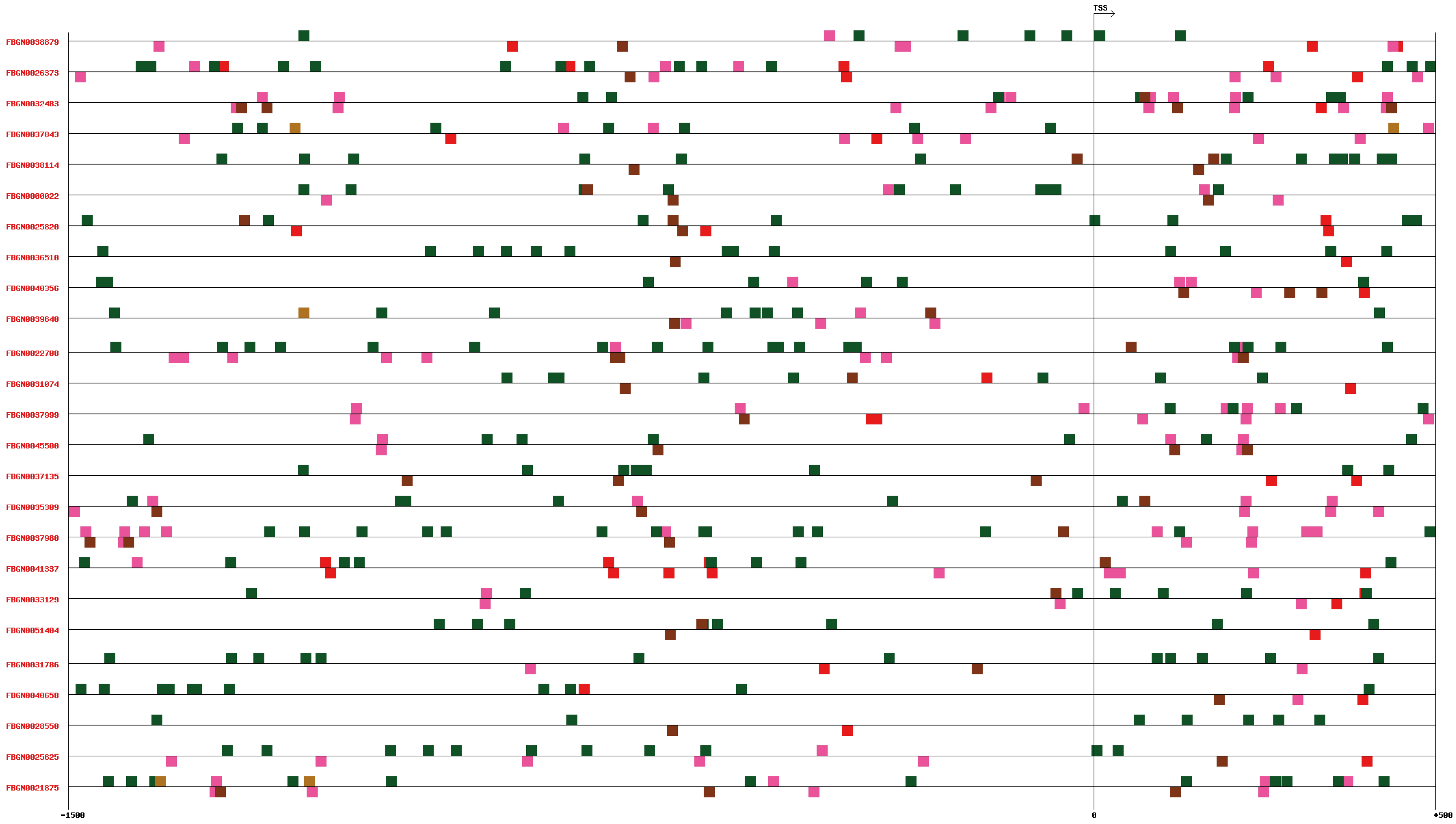

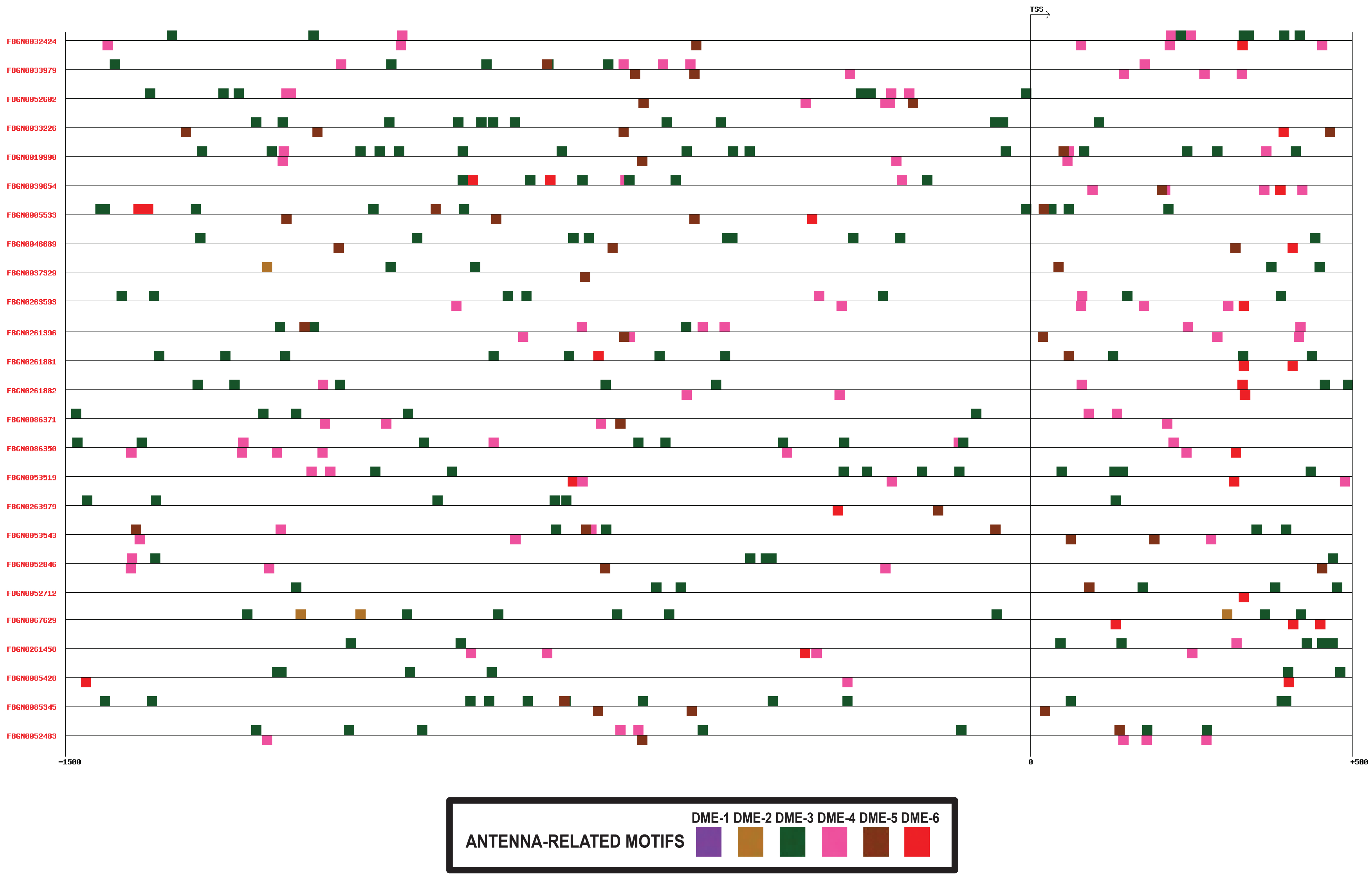
TSS
FBGN0032424
FBGN0033979
FBGN0052602
FBGN0033226
FBGN0019990
FBGN0039654
FBGN0005533
FBGN0046689
FBGN0037329
FBGN0263593
FBGN0261396
FBGN0261881
FBGN0261882
FBGN0086371
FBGN0086350
FBGN0053519
FBGN0263979
FBGN0053543
FBGN0052846
FBGN0052712
FBGN0067629
FBGN0261458
FBGN0085428
FBGN0085345
FBGN0052483
-1500
0
+500
ANTENNA-RELATED MOTIFS
DME-1 DME-2 DME-3 DME-4 DME-5 DME-6
