# Supplementary material for: A Set of Structural Features Defines the Cis-Regulatory Modules of Antenna-Expressed Genes in Drosophila melanogaster
Source: PLoS One. 2014 Aug 25;9(8):e104342. doi: 10.1371/journal.pone.0104342 (PMC4143197; doi:10.1371/journal.pone.0104342)

Figure S3: Conservation of structural features between the regulatory region of *D. melanogaster Gr22b* and the regulatory regions of orthologs across the *Drosophila* lineage. Colored squares represent antenna-related motifs. Squares above or under the black line indicate motifs on the plus or minus strand, respectively. Red cross means either that the respective regulatory region does not contain conserved features or that there is no such ortholog. The phylogenetic tree is based on the tree reported in [46].

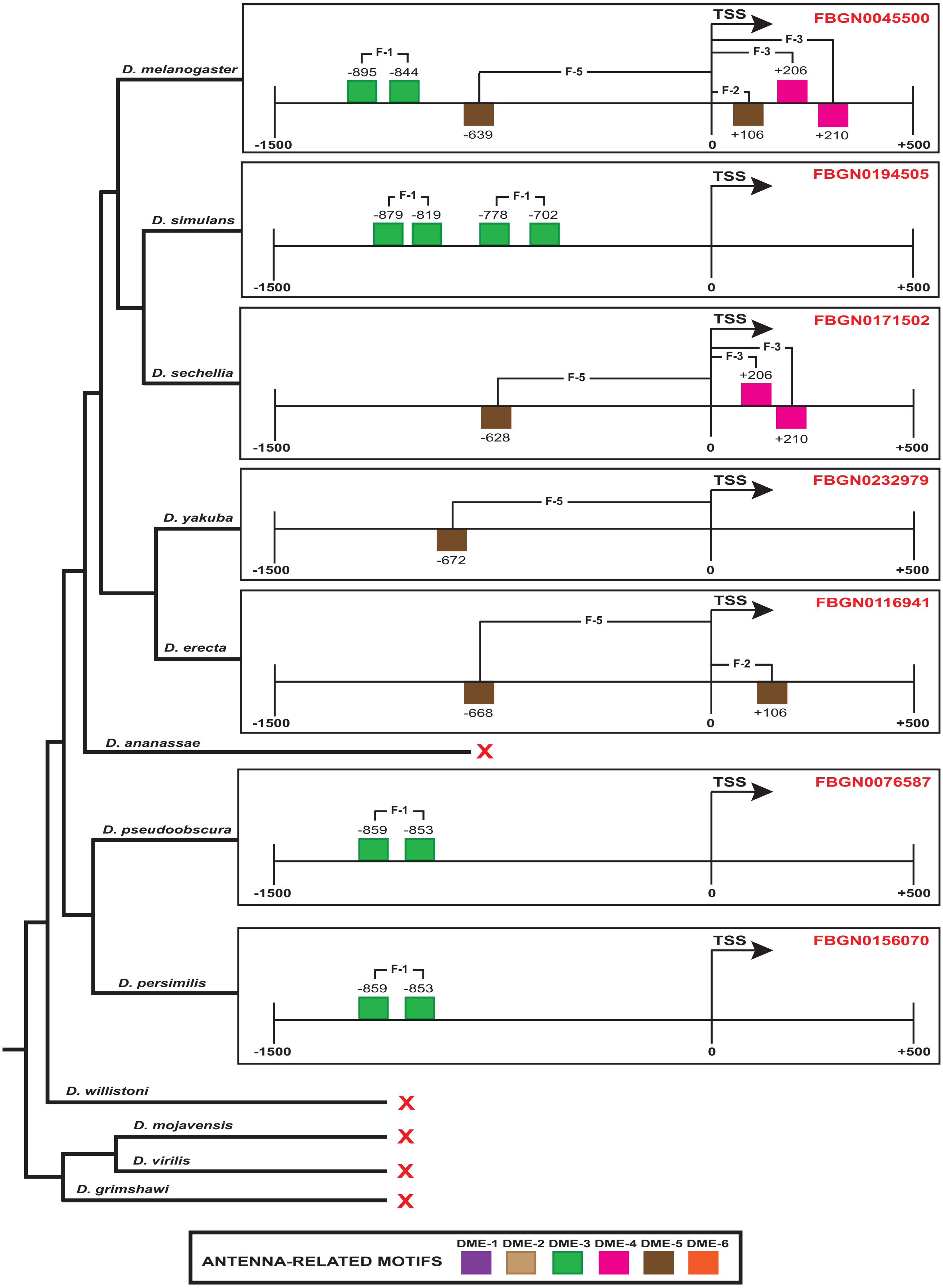

Supplement: Figure S3 — Conservation of structural features between the regulatory region of D. melanogaster Gr22b and the regulatory regions of orthologs across the Drosophila lineage. (PDF) [file pone.0104342.s003.pdf]
