# Supplementary material for: A Set of Structural Features Defines the Cis-Regulatory Modules of Antenna-Expressed Genes in Drosophila melanogaster
Source: PLoS One. 2014 Aug 25;9(8):e104342. doi: 10.1371/journal.pone.0104342 (PMC4143197; doi:10.1371/journal.pone.0104342)
Supplement: Figure S4 — The 50 highest-scoring regulatory regions in C. elegans . (PDF) [file pone.0104342.s004.pdf]

Figure S4: The 50 highest-scoring regulatory regions in *C. elegans*. Colored squares represent muscle-related motifs. Squares above or under the black line indicate motifs on the plus or minus strand, respectively.

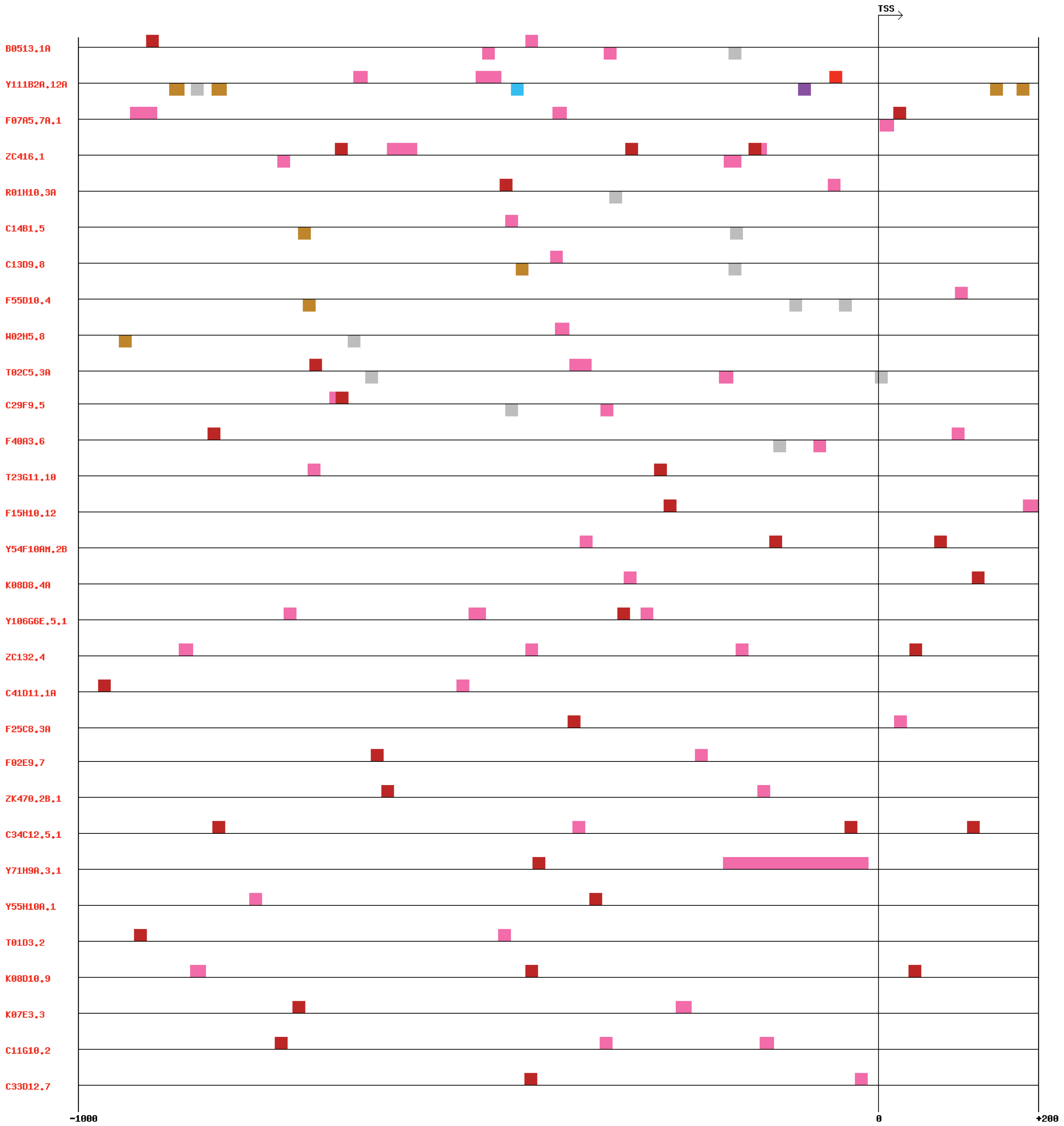

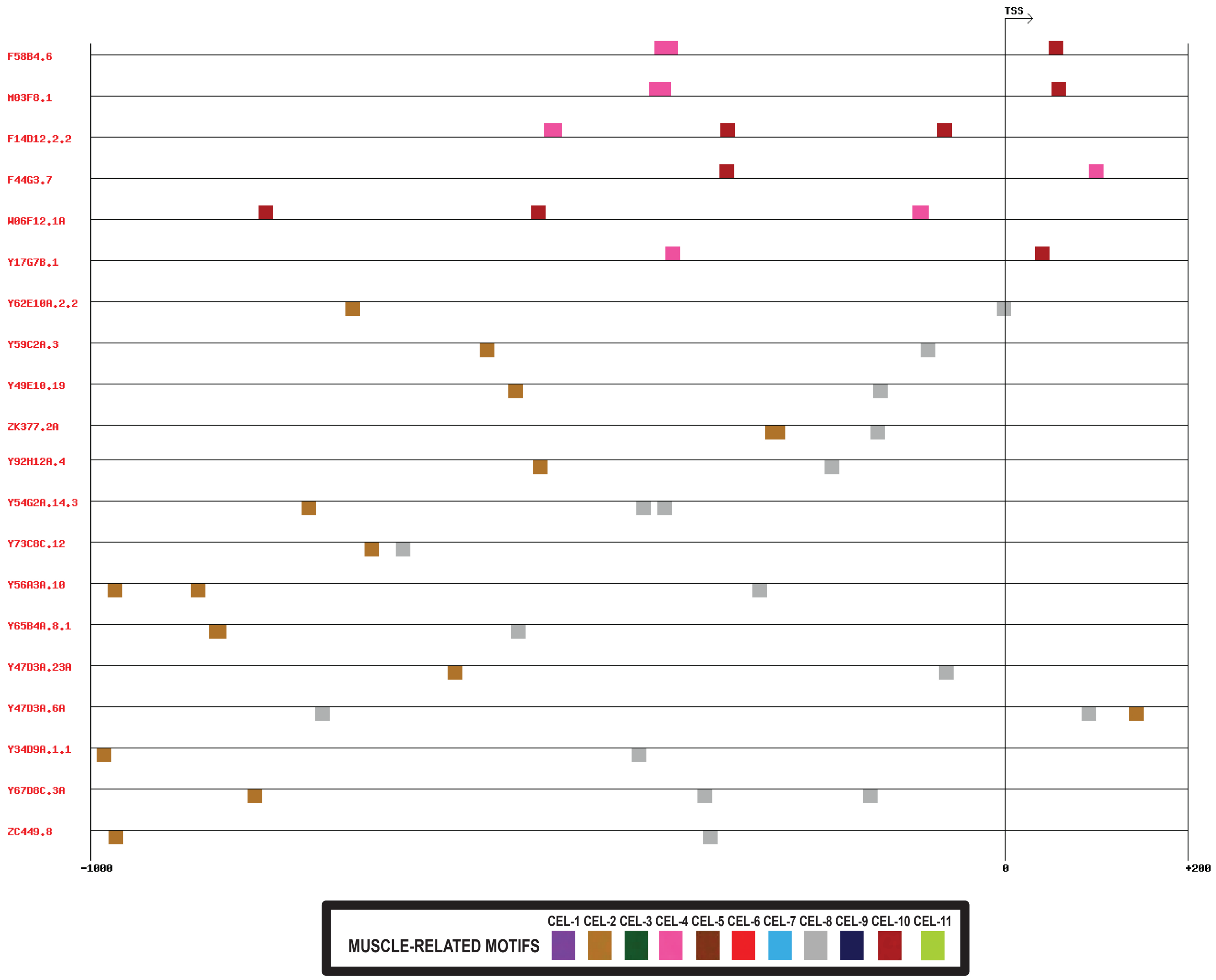
TSS
F58B4.6
M03F8.1
F14D12.2.2
F44G3.7
W06F12.1A
Y17G7B.1
Y62E10A.2.2
Y59C2A.3
Y49E10.19
ZK377.2A
Y92H12A.4
Y54G2A.14.3
Y73C8C.12
Y56A3A.10
Y65B4A.8.1
Y47D3A.23A
Y47D3A.6A
Y34D9A.1.1
Y67D8C.3A
ZC449.8
-1000
0
+200
MUSCLE-RELATED MOTIFS
CEL-1 CEL-2 CEL-3 CEL-4 CEL-5 CEL-6 CEL-7 CEL-8 CEL-9 CEL-10 CEL-11
