# Supplementary material for: A Set of Structural Features Defines the Cis-Regulatory Modules of Antenna-Expressed Genes in Drosophila melanogaster
Source: PLoS One. 2014 Aug 25;9(8):e104342. doi: 10.1371/journal.pone.0104342 (PMC4143197; doi:10.1371/journal.pone.0104342)

**Figure S5: Detailed architecture of two *C. elegans* regulatory regions uncovered by our method and previously reported [13].** Colored squares represent muscle-related motifs. Squares above or under the black line indicate motifs on the plus or minus strand, respectively.

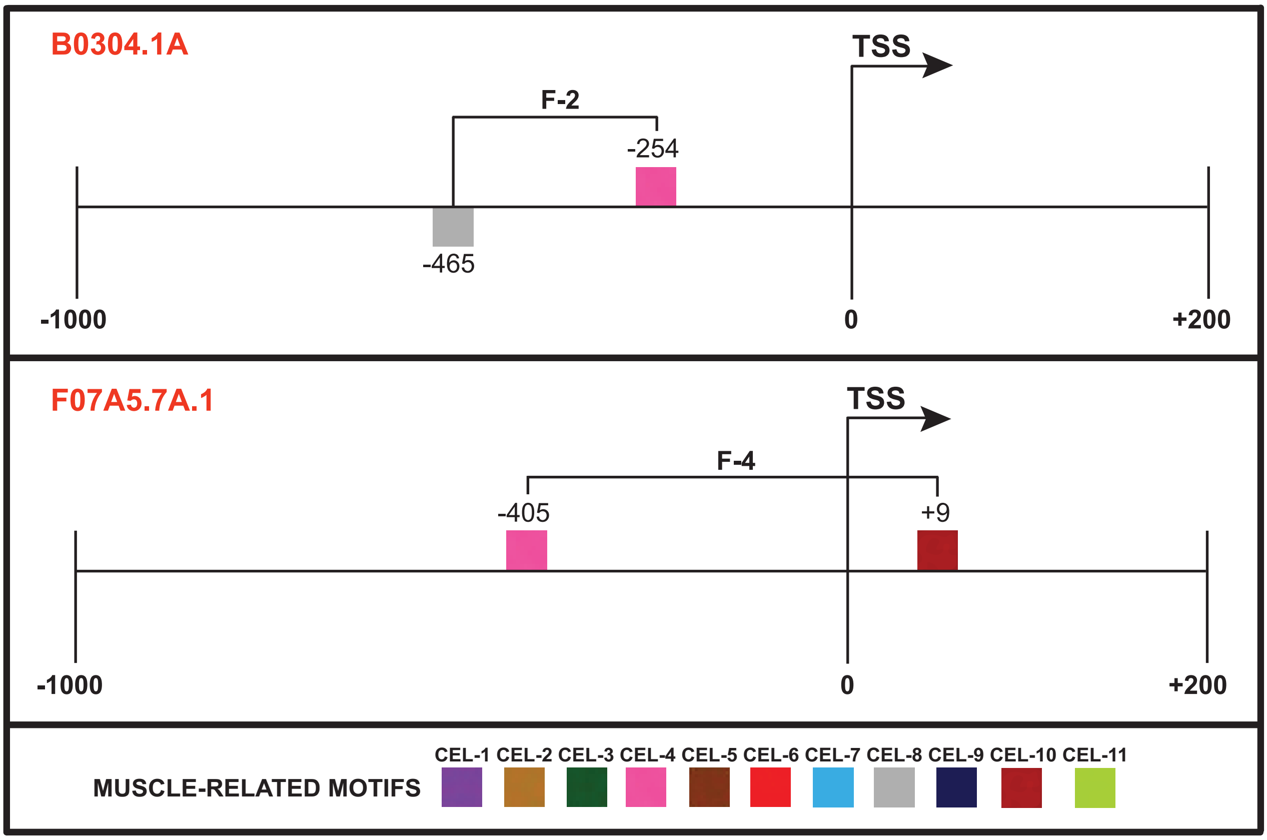

Supplement: Figure S5 — Detailed architecture of two C. elegans regulatory regions uncovered by our method and previously reported [13]. (PDF) [file pone.0104342.s005.pdf]
