# Supplementary material for: A Set of Structural Features Defines the Cis-Regulatory Modules of Antenna-Expressed Genes in Drosophila melanogaster
Source: PLoS One. 2014 Aug 25;9(8):e104342. doi: 10.1371/journal.pone.0104342 (PMC4143197; doi:10.1371/journal.pone.0104342)
Supplement: Table S1 — Gene ontology terms for the 1000 genes (excluding genes in the initial sets) with the highest-scoring regulatory regions. (PDF) [file pone.0104342.s006.pdf]

Table S1: Gene ontology terms for the 1000 genes (excluding genes in the initial sets) with the highest-scoring regulatory regions. "Count" is the number of genes with each annotation. Both uncorrected and multiple testing-corrected *p*-values are included.

Gene Ontology Term	Count	*P*-Value	Benjamini
RNA degradation	12	1.6E-3	1.3E-1
transcription	45	2.4E-3	9.7E-1
FBOX	9	2.4E-3	3.8E-1
dioxygenase	5	3.4E-3	5.7E-1
transcription, DNA-dependent	18	3.5E-3	9.3E-1
RNA biosynthetic process	18	4.4E-3	8.9E-1
GPI anchor metabolic process	7	4.4E-3	8.2E-1
Cyclin-like F-box	9	4.7E-3	9.9E-1
nucleoplasm part	28	4.8E-3	8.1E-1
endomembrane system	26	5.1E-3	5.9E-1
nucleoplasm	30	5.2E-3	4.5E-1
histone modification	10	6.3E-3	8.6E-1
covalent chromatin modification	10	6.3E-3	8.6E-1
organelle lumen	53	6.4E-3	4.3E-1
intracellular organelle lumen	53	6.4E-3	4.3E-1
vesicle-mediated transport	38	6.4E-3	8.1E-1
membrane-enclosed lumen	54	6.9E-3	3.8E-1
bristle morphogenesis	9	7.7E-3	8.2E-1
transcription from RNA polymerase II promoter	14	8.0E-3	7.9E-1
chromatin modification	15	9.2E-3	8.0E-1
