# Supplementary material for: A Set of Structural Features Defines the Cis-Regulatory Modules of Antenna-Expressed Genes in Drosophila melanogaster
Source: PLoS One. 2014 Aug 25;9(8):e104342. doi: 10.1371/journal.pone.0104342 (PMC4143197; doi:10.1371/journal.pone.0104342)
Supplement: Table S2 — Description of 50 D. melanogaster genes with high scoring regulatory regions. (PDF) [file pone.0104342.s007.pdf]

Table S2: Description of 50 *D. melanogaster* genes with high scoring regulatory regions. For each gene, FlyBase ID, symbol, score and description of biological function are shown. Bolded lines correspond to genes whose regulatory regions are detailed in figure 6.

FlyBase ID	Symbol	Score	Biological Function
**FBGN0038879**	**Dmel\CG17298**	**0.07**	**unknown molecular function and biological process**
FBGN0026373	Dmel\RpII33	0.07	involved in transcription from RNA polymerase II promoter and cellular response to heat
FBGN0032483	Dmel\CG15482	0.07	unknown molecular function and biological process
FBGN0037843	Dmel\CG4511	0.06	unknown biological process
FBGN0038114	Dmel\CG11670	0.06	involved in proteolysis
**FBGN0000022**	**Dmel\ac**	**0.06**	**involved in neuroblast fate commitment and sensory organ development**
FBGN0025820	Dmel\JTBR	0.06	unknown molecular function and biological process
FBGN0036510	Dmel\CG7427	0.06	unknown molecular function and biological processes
FBGN0040356	Dmel\CG12498	0.06	involved in histone modification and DNA-dependent transcription
FBGN0039640	Dmel\CG14516	0.06	involved in proteolysis
**FBGN0022708**	**Dmel\Adk2**	**0.06**	**involved in neurogenesis and ADP biosynthetic process**
FBGN0031074	Dmel\skpE	0.06	involved in ubiquitin-dependent protein catabolic process
FBGN0037999	Dmel\CG4860	0.06	involved in fatty acid beta-oxidation
**FBGN0045500**	**Dmel\Gr22b**	**0.06**	**involved in detection of chemical stimulus involved in sensory**
FBGN0037135	Dmel\CG7414	0.06	involved in ribosome assembly and regulation of translation
FBGN0035309	Dmel\CG15879	0.06	unknown molecular function and biological process
FBGN0037980	Dmel\CG3313	0.06	unknown molecular function and biological process
FBGN0041337	Dmel\Cyp309a2	0.06	involved in oxidation-reduction process
FBGN0033129	Dmel\Tsp42Eh	0.06	high expression levels in larval carcass
FBGN0051404	Dmel\CG31404	0.06	moderate expression levels in adult testis
FBGN0031786	Dmel\CG13989	0.06	unknown molecular function and biological process
FBGN0040658	Dmel\CG13516	0.06	unknown molecular function and biological process
FBGN0028550	Dmel\Atf3	0.06	involved in nervous system development and lipid homeostasis
FBGN0025625	Dmel\Sik2	0.06	involved in protein phosphorylation and response to starvation
FBGN0021875	Dmel\Zfrp8	0.06	involved in cell proliferation, embryonic hemopoiesis and somatic stem cell division
FBGN0032424	Dmel\CG17010	0.06	involved in D-ribose metabolic process
FBGN0033979	Dmel\Cyp6a19	0.06	involved in oxidation-reduction process
FBGN0052602	Dmel\Muc12Ea	0.06	involved in neurogenesis, chorion-containing eggshell formation

Continued on next page

Table S2: *Continued from previous page*

FlyBase ID	Symbol	Score	Biological Function
FBGN0033226	Dmel\CG1882	0.06	unknown biological process
FBGN0019990	Dmel\Gcn2	0.06	involved in mRNA splicing, via spliceosome and regulation of translation
FBGN0039654	Dmel\Brd8	0.06	involved in negative regulation of gene expression
FBGN0005533	Dmel\RpS17	0.06	involved in translational elongation, translation and ribosomal small subunit assembly
FBGN0046689	Dmel\Takl1	0.06	involved in protein phosphorylation
FBGN0037329	Dmel\CG12162	0.06	unknown biological process
FBGN0263593	Dmel\Lpin	0.05	involved in triglyceride biosynthetic process and imaginal disc-derived wing vein specification
FBGN0261396	Dmel\Rpn3	0.05	involved in proteolysis and regulation of protein catabolic process
FBGN0261881	Dmel\l(2)35Be	0.05	phenotype manifested in mesothoracic tergum
FBGN0261882	Dmel\l(2)35Bc	0.05	involved in tRNA modification and neurogenesis
FBGN0086371	Dmel\poly	0.05	involved in insulin receptor signaling pathway and oocyte microtubule cytoskeleton polarization
FBGN0086350	Dmel\tef	0.05	involved in male meiosis chromosome segregation and synapsis
FBGN0053519	Dmel\Unc-89	0.05	involved in sarcomere organization and adult somatic muscle development
FBGN0263979	Dmel\Caf1	0.05	involved in neuron differentiation and system development
FBGN0053543	Dmel\CG33543	0.05	involved in cell adhesion
FBGN0052846	Dmel\CG32846	0.05	peak expression levels observed during early pupal stages
FBGN0052712	Dmel\CG32712	0.05	moderate expression levels in adult testis
FBGN0067629	Dmel\CG33332	0.05	moderate expression levels in adult ovary
FBGN0261458	Dmel\capt	0.05	involved in sensory organ development and compound eye photoreceptor development
FBGN0085428	Dmel\Nox	0.05	involved in oxidation-reduction process
FBGN0085345	Dmel\CG34316	0.05	unknown biological process
FBGN0052483	Dmel\CG32483	0.05	involved in proteolysis
