# Supplementary material for: A Set of Structural Features Defines the Cis-Regulatory Modules of Antenna-Expressed Genes in Drosophila melanogaster
Source: PLoS One. 2014 Aug 25;9(8):e104342. doi: 10.1371/journal.pone.0104342 (PMC4143197; doi:10.1371/journal.pone.0104342)
Supplement: Table S3 — Predicted motifs in regulatory regions of muscle-expressed genes. (PDF) [file pone.0104342.s008.pdf]

Table S3: Predicted motifs in regulatory regions of muscle-expressed genes. For each motif, the identifier, logo and over-representation index (ORI) are shown. The known regulatory motif, TOMTOM p-value, and citations are also given for motifs that matched already identified motifs.

ID	Logo	ORI	Comment	Citations
CEL-1	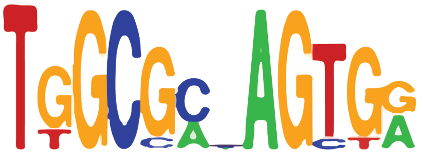	2.373	-	-
CEL-2	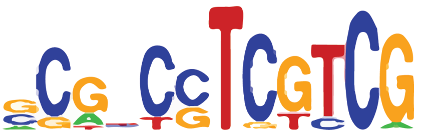	3.503	-	-
CEL-3	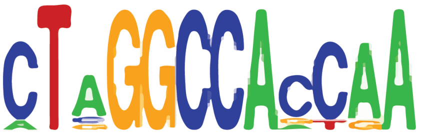	2.904	-	-
CEL-4	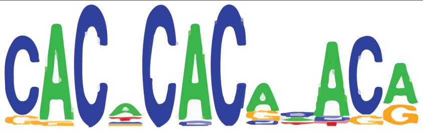	6.935	DAF-12 (7.55e-05)	[32]
CEL-5	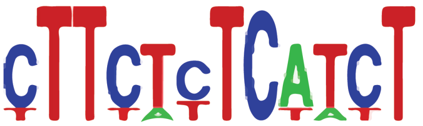	3.145	-	-
CEL-6	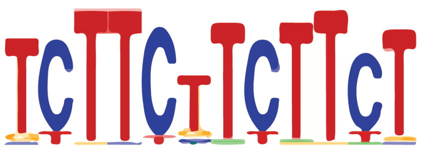	2.215	EOR-1 (1.91e-03)	[33]
CEL-7	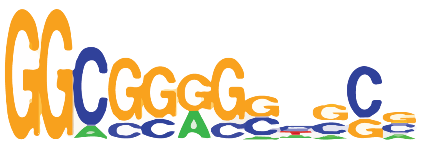	7.093	-	-
CEL-8	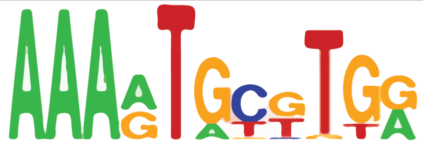	2.878	-	-
CEL-9	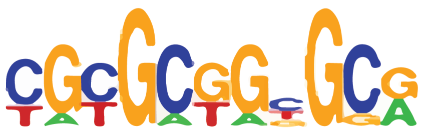	5.151	DPY-27 (9.70e-03)	[34]
CEL-10	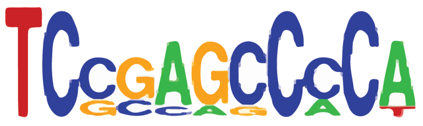	2.090	-	-
CEL-11	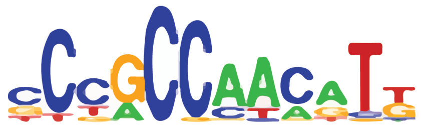	3.967	-	-
