# Supplementary material for: A Set of Structural Features Defines the Cis-Regulatory Modules of Antenna-Expressed Genes in Drosophila melanogaster
Source: PLoS One. 2014 Aug 25;9(8):e104342. doi: 10.1371/journal.pone.0104342 (PMC4143197; doi:10.1371/journal.pone.0104342)
Supplement: Table S4 — The set of structural features that best describe the regulatory regions of muscle-expressed genes in C. elegans . (PDF) [file pone.0104342.s009.pdf]

Table S4: The set of structural features that best describe the regulatory regions of muscle-expressed genes in *C. elegans*. For each feature, the relationship between motifs within the feature, and the Kullback-Leibler weight are shown. Colored squares represent muscle-related motifs. Squares above or under the black line indicate motifs on the plus or minus strand, respectively.

Illustration	Description and Weights
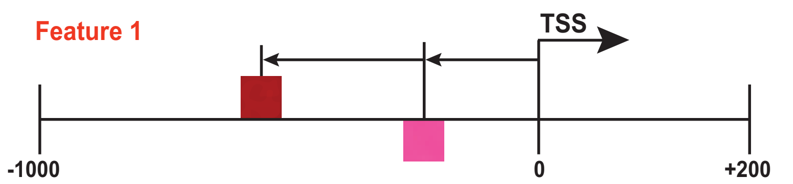	CEL-4 is positioned downstream from CEL-10 on opposite strands (0.1)
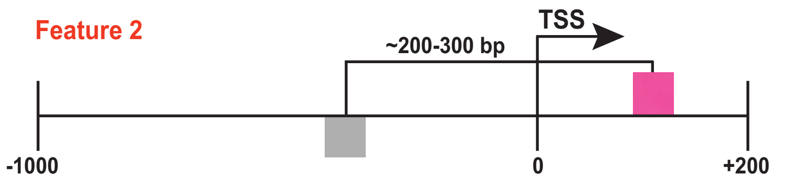	CEL-4 is positioned at ~200-300 bp from CEL-8 on opposite strands (0.09)
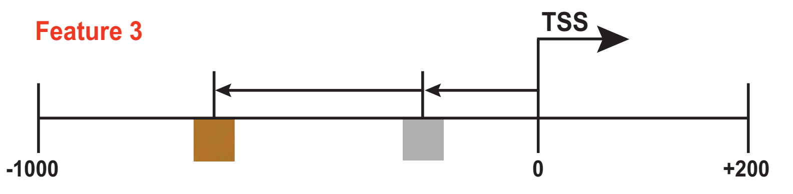	CEL-8 is positioned downstream from CEL-2 on the minus strand (0.13)
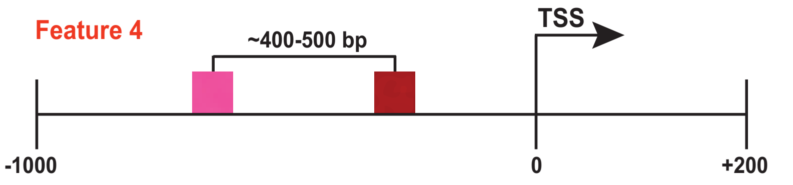	CEL-10 is positioned at ~400-500 bp downstream from CEL-4 on the plus strand (0.14)
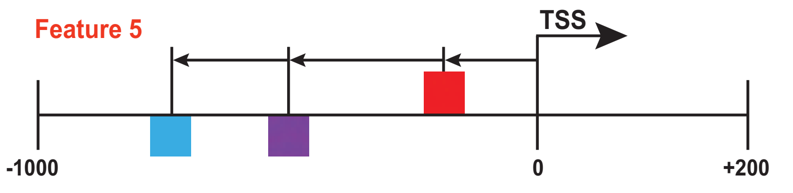	CEL-6 is positioned downstream from CEL-1 and CEL-7 on opposite strands (0.06)
MUSCLE-RELATED MOTIFS 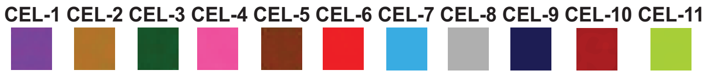	
