# Supplementary material for: A Set of Structural Features Defines the Cis-Regulatory Modules of Antenna-Expressed Genes in Drosophila melanogaster
Source: PLoS One. 2014 Aug 25;9(8):e104342. doi: 10.1371/journal.pone.0104342 (PMC4143197; doi:10.1371/journal.pone.0104342)
Supplement: Table S5 — Description of 50 C. elegans genes with high scoring regulatory regions. (PDF) [file pone.0104342.s010.pdf]

Table S5: Description of 50 *C. elegans* genes with high scoring regulatory regions. For each gene, the name (transcript), score and biological function are shown.

Gene Name (Transcript)	Score	Biological Function
lin-66 (B0513.1A)	0.33	expressed in body wall and vulval muscles
Y111B2A.12 (Y111B2A.12A)	0.28	involved in nematode larval development and positive regulation of multicellular organism growth
unc-15 (F07A5.7A.1)	0.24	UNC-15 protein physically interacts with an isoform of myosin heavy chain (MHC) in striated muscle
madf-9 (ZC416.1)	0.24	no gene ontology data available
cor-1 (R01H10.3A)	0.23	encodes an ortholog of the actin-binding protein coronin
dph-1 (C14B1.5)	0.22	paralogous to *S. cerevisiae* DPH2/YKL191W, a protein component of diphtamide synthesis
ncx-9 (C13D9.8)	0.22	encodes a putative Na[+]/Ca[2+] exchanger of uncertain stoichiometry and affinity
F55D10.4 (F55D10.4)	0.22	no gene ontology data available
W02H5.8 (W02H5.8)	0.22	involved in glycerol metabolic process
igcm-3 (T02C5.3A)	0.19	encodes protein containing an immunoglobulin-like domain
C29F9.5 (C29F9.5)	0.19	involved in regulation of transcription
F40A3.6 (F40A3.6)	0.19	no gene ontology data available
T23G11.10 (T23G11.10)	0.14	involved in germline cell cycle switching and regulation of cell proliferation
F15H10.12 (F15H10.12)	0.14	no gene ontology data available
feh-1 (Y54F10AM.2B)	0.14	expressed in the neuromuscular structures of the pharynx
K08D8.4 (K08D8.4A)	0.14	no gene ontology data available
ced-12 (Y106G6E.5.1)	0.14	required for phagocytotic engulfment of apoptotic cells
ZC132.4 (ZC132.4)	0.14	no gene ontology data available
eri-6 (C41D11.1A)	0.14	involved in reproduction
unc-80 (F25C8.3A)	0.14	unc-80::gfp reporter fusion is expressed in sensory and motoneurons
F02E9.7 (F02E9.7)	0.14	molecular function in hydrolase activity
ZK470.2 (ZK470.2B.1)	0.14	no gene ontology data available
rsu-1 (C34C12.5.1)	0.14	no gene ontology data available
sto-4 (Y71H9A.3.1)	0.14	STOmatin plays a role in membrane
vha-19 (Y55H10A.1)	0.14	VHA-19 is predicted to help carry protons from the cytosol to a-subunits for transmembrane export
hlh-34 (T01D3.2)	0.14	involved in regulation of transcription and signal transduction
K08D10.9 (K08D10.9)	0.14	no gene ontology data available
dao-3 (K07E3.3)	0.14	dao-3 promoter gfp fusion is expressed in larvae in the hypodermis and in the nervous system

Continued on next page

Table S5: *Continued from previous page*

Gene Name (Transcript)	Score	Biological Function
C11G10.2 (C11G10.2)	0.14	no gene ontology data available
ceh-30 (C33D12.7)	0.14	CEH-30 functions as a key regulator of sex-specific apoptosis
F58B4.6 (F58B4.6)	0.14	no gene ontology data available
M03F8.1 (M03F8.1)	0.14	no gene ontology data available
unc-97 (F14D12.2.2)	0.14	involved in assembling of muscle adherens junctions and mechanosensory functions of touch neurons
F44G3.7 (F44G3.7)	0.14	no gene ontology data available
lit-1 (W06F12.1A)	0.14	expressed in most embryonic and larval cells, including the amphid sheath glia
acbp-6 (Y17G7B.1)	0.14	ACBP-6::GFP is uniquely expressed in specific head, body and tail neurons
Y62E10A.2 (Y62E10A.2.2)	0.13	encodes an ortholog of Pop7 (protein subunit shared by the endoribonuclease RNase MRP)
Y59C2A.3 (Y59C2A.3)	0.13	no gene ontology data available
ani-1 (Y49E10.19)	0.13	ANI-1 plays a role in cuticle formation, coordinated locomotion, vulval development, and male tail formation
sax-3 (ZK377.2A)	0.13	SAX-3 is required to confine migrating sex myoblasts to the ventral muscle quadrants
Y92H12A.4 (Y92H12A.4)	0.13	no gene ontology data available
clec-83 (Y54G2A.14.3)	0.13	involved in carbohydrate binding
Y73C8C.12 (Y73C8C.12)	0.13	no gene ontology data available
fbxb-22 (Y56A3A.10)	0.13	encodes a protein containing an F-box
Y65B4A.8 (Y65B4A.8.1)	0.13	involved in biosynthetic process and coenzyme A biosynthetic process
gly-9 (Y47D3A.23A)	0.13	involved in carbohydrate metabolic process
tra-1 (Y47D3A.6A)	0.13	TRA-1 is expressed in hermaphrodites and males
mrpl-38 (Y34D9A.1.1)	0.13	involved in nematode larval development
Y67D8C.3 (Y67D8C.3A)	0.13	involved in oviposition and reproduction
ZC449.8 (ZC449.8)	0.13	no gene ontology data available
