# Supplementary material for: A Set of Structural Features Defines the Cis-Regulatory Modules of Antenna-Expressed Genes in Drosophila melanogaster
Source: PLoS One. 2014 Aug 25;9(8):e104342. doi: 10.1371/journal.pone.0104342 (PMC4143197; doi:10.1371/journal.pone.0104342)
Supplement: Table S6 — FlyBase IDs of 90 antenna-expressed genes in the “motif-prediction” set. (PDF) [file pone.0104342.s011.pdf]

Table S6: FlyBase IDs of 90 antenna-expressed genes in the "motif-prediction" set.

FBGN0038531	FBGN0038734	FBGN0041624	FBGN0034487	FBGN0041621
FBGN0037590	FBGN0033463	FBGN0030868	FBGN0038958	FBGN0030005
FBGN0031289	FBGN0052448	FBGN0036062	FBGN0017457	FBGN0034224
FBGN0036936	FBGN0034768	FBGN0085325	FBGN0041250	FBGN0003450
FBGN0038114	FBGN0031258	FBGN0036859	FBGN0036938	FBGN0004898
FBGN0026392	FBGN0035865	FBGN0037345	FBGN0052250	FBGN0035475
FBGN0004400	FBGN0033140	FBGN0039325	FBGN0034565	FBGN0039510
FBGN0004832	FBGN0033413	FBGN0040261	FBGN0029853	FBGN0033628
FBGN0040849	FBGN0031479	FBGN0050101	FBGN0031589	FBGN0026389
FBGN0052797	FBGN0041622	FBGN0264954	FBGN0052801	FBGN0262123
FBGN0032085	FBGN0051718	FBGN0031529	FBGN0031059	FBGN0038309
FBGN0003346	FBGN0030016	FBGN0034770	FBGN0037025	FBGN0022724
FBGN0026386	FBGN0261401	FBGN0051717	FBGN0024891	FBGN0033072
FBGN0032181	FBGN0001967	FBGN0039202	FBGN0030244	FBGN0032127
FBGN0036019	FBGN0050051	FBGN0037576	FBGN0041241	FBGN0261380
FBGN0037000	FBGN0034865	FBGN0037399	FBGN0037501	FBGN0010786
FBGN0040253	FBGN0041625	FBGN0032189	FBGN0015553	FBGN0033529
FBGN0003480	FBGN0011787	FBGN0032822	FBGN0053475	FBGN0033931
