# Supplementary material for: A Set of Structural Features Defines the Cis-Regulatory Modules of Antenna-Expressed Genes in Drosophila melanogaster
Source: PLoS One. 2014 Aug 25;9(8):e104342. doi: 10.1371/journal.pone.0104342 (PMC4143197; doi:10.1371/journal.pone.0104342)
Supplement: Table S7 — FlyBase IDs of 44 antenna-expressed genes in the “feature-generation” set. (PDF) [file pone.0104342.s012.pdf]

Table S7: FlyBase IDs of 44 antenna-expressed genes in the "feature-generation" set.

FBGN0030234	FBGN0044811	FBGN0052405	FBGN0051075	FBGN0051019
FBGN0052277	FBGN0024249	FBGN0050259	FBGN0036219	FBGN0262685
FBGN0041623	FBGN0050272	FBGN0026373	FBGN0035468	FBGN0024947
FBGN0036212	FBGN0037685	FBGN0038798	FBGN0010651	FBGN0038404
FBGN0031943	FBGN0028946	FBGN0033209	FBGN0039879	FBGN0036638
FBGN0036414	FBGN0036923	FBGN0050044	FBGN0033362	FBGN0037411
FBGN0036009	FBGN0036195	FBGN0027073	FBGN0036628	FBGN0037934
FBGN0053757	FBGN0013749	FBGN0036240	FBGN0031694	FBGN0039009
FBGN0037519	FBGN0035435	FBGN0036078	FBGN0033501	
