# Supplementary material for: A Set of Structural Features Defines the Cis-Regulatory Modules of Antenna-Expressed Genes in Drosophila melanogaster
Source: PLoS One. 2014 Aug 25;9(8):e104342. doi: 10.1371/journal.pone.0104342 (PMC4143197; doi:10.1371/journal.pone.0104342)
Supplement: Table S8 — FlyBase IDs of 90 antenna-expressed genes in the “model-build” set. (PDF) [file pone.0104342.s013.pdf]

Table S8: FlyBase IDs of 90 antenna-expressed genes in the "model-build" set.

FBGN0036828	FBGN0038452	FBGN0024352	FBGN0085295	FBGN0031998
FBGN0035168	FBGN0044511	FBGN0035256	FBGN0032684	FBGN0004404
FBGN0053208	FBGN0034769	FBGN0027348	FBGN0026395	FBGN0034906
FBGN0039673	FBGN0034106	FBGN0024432	FBGN0039454	FBGN0032428
FBGN0031324	FBGN0035031	FBGN0032052	FBGN0031854	FBGN0035002
FBGN0036206	FBGN0034473	FBGN0031209	FBGN0025558	FBGN0032877
FBGN0030389	FBGN0038814	FBGN0038602	FBGN0031725	FBGN0000137
FBGN0039319	FBGN0047330	FBGN0031791	FBGN0026385	FBGN0040256
FBGN0038916	FBGN0085326	FBGN0030804	FBGN0036239	FBGN0037989
FBGN0038727	FBGN0031668	FBGN0038799	FBGN0035604	FBGN0053658
FBGN0045502	FBGN0033043	FBGN0039324	FBGN0003462	FBGN0033357
FBGN0030598	FBGN0085424	FBGN0052704	FBGN0038397	FBGN0013812
FBGN0032406	FBGN0039201	FBGN0032211	FBGN0030204	FBGN0034176
FBGN0037726	FBGN0035286	FBGN0026398	FBGN0035742	FBGN0039551
FBGN0038203	FBGN0028963	FBGN0034766	FBGN0032949	FBGN0038350
FBGN0035085	FBGN0085260	FBGN0015271	FBGN0034692	FBGN0035887
FBGN0039385	FBGN0053289	FBGN0030669	FBGN0034909	FBGN0003382
FBGN0036764	FBGN0026397	FBGN0051216	FBGN0036143	FBGN0030395
